# Evaluation of Plasma Phosphorylated Tau217 for Differentiation Between Alzheimer Disease and Frontotemporal Lobar Degeneration Subtypes Among Patients With Corticobasal Syndrome

**DOI:** 10.1001/jamaneurol.2023.0488

**Published:** 2023-04-03

**Authors:** Lawren VandeVrede, Renaud La Joie, Elisabeth H. Thijssen, Breton M. Asken, Stephanie A. Vento, Torie Tsuei, Suzanne L. Baker, Yann Cobigo, Corrina Fonseca, Hilary W. Heuer, Joel H. Kramer, Peter A. Ljubenkov, Gil D. Rabinovici, Julio C. Rojas, Howie J. Rosen, Adam M. Staffaroni, Brad F. Boeve, Brad C. Dickerson, Murray Grossman, Edward D. Huey, David J. Irwin, Irene Litvan, Alexander Y. Pantelyat, Maria Carmela Tartaglia, Jeffrey L. Dage, Adam L. Boxer

**Affiliations:** 1 Department of Neurology, Memory and Aging Center, University of California San Francisco Weill Institute for Neurosciences, University of California, San Francisco; 2 Lawrence Berkeley National Laboratory, Berkeley, California; 3 Neurochemistry Laboratory, Department of Clinical Chemistry, Vrije Universiteit Amsterdam, Amsterdam University Medical Center, Amsterdam, the Netherlands; 4 Fixel Institute for Neurological Disease, Department of Clinical and Healthy Psychology, University of Florida, Gainesville; 5 Associate Editor, *JAMA Neurology*; 6 Department of Neurology, Mayo Clinic, Rochester, Minnesota; 7 Frontotemporal Disorders Unit, Massachusetts General Hospital, Boston; 8 Penn Frontotemporal Degeneration Center, Perelman School of Medicine, University of Pennsylvania, Philadelphia; 9 Department of Psychiatry, Columbia University, New York, New York; 10 Department of Neurology, Columbia University, New York, New York; 11 Department of Neurology, University of California, San Diego; 12 Department of Neurology, Johns Hopkins University School of Medicine, Baltimore, Maryland; 13 Tanz Centre for Research in Neurodegenerative Diseases, University of Toronto, Toronto, Ontario, Canada; 14 Department of Neurology, Indiana University School of Medicine, Indianapolis

## Abstract

**Importance:**

Plasma phosphorylated tau217 (p-tau217), a biomarker of Alzheimer disease (AD), is of special interest in corticobasal syndrome (CBS) because autopsy studies have revealed AD is the driving neuropathology in up to 40% of cases. This differentiates CBS from other 4-repeat tauopathy (4RT)–associated syndromes, such as progressive supranuclear palsy Richardson syndrome (PSP-RS) and nonfluent primary progressive aphasia (nfvPPA), where underlying frontotemporal lobar degeneration (FTLD) is typically the primary neuropathology.

**Objective:**

To validate plasma p-tau217 against positron emission tomography (PET) in 4RT-associated syndromes, especially CBS.

**Design, Setting, and Participants:**

This multicohort study with 6, 12, and 24-month follow-up recruited adult participants between January 2011 and September 2020 from 8 tertiary care centers in the 4RT Neuroimaging Initiative (4RTNI). All participants with CBS (n = 113), PSP-RS (n = 121), and nfvPPA (n = 39) were included; other diagnoses were excluded due to rarity (n = 29). Individuals with PET-confirmed AD (n = 54) and PET-negative cognitively normal control individuals (n = 59) were evaluated at University of California San Francisco. Operators were blinded to the cohort.

**Main Outcome and Measures:**

Plasma p-tau217, measured by Meso Scale Discovery electrochemiluminescence, was validated against amyloid-β (Aβ) and flortaucipir (FTP) PET. Imaging analyses used voxel-based morphometry and bayesian linear mixed-effects modeling. Clinical biomarker associations were evaluated using longitudinal mixed-effect modeling.

**Results:**

Of 386 participants, 199 (52%) were female, and the mean (SD) age was 68 (8) years. Plasma p-tau217 was elevated in patients with CBS with positive Aβ PET results (mean [SD], 0.57 [0.43] pg/mL) or FTP PET (mean [SD], 0.75 [0.30] pg/mL) to concentrations comparable to control individuals with AD (mean [SD], 0.72 [0.37]), whereas PSP-RS and nfvPPA showed no increase relative to control. Within CBS, p-tau217 had excellent diagnostic performance with area under the receiver operating characteristic curve (AUC) for Aβ PET of 0.87 (95% CI, 0.76-0.98; *P* < .001) and FTP PET of 0.93 (95% CI, 0.83-1.00; *P* < .001). At baseline, individuals with CBS-AD (n = 12), defined by a PET-validated plasma p-tau217 cutoff 0.25 pg/mL or greater, had increased temporoparietal atrophy at baseline compared to individuals with CBS-FTLD (n = 39), whereas longitudinally, individuals with CBS-FTLD had faster brainstem atrophy rates. Individuals with CBS-FTLD also progressed more rapidly on a modified version of the PSP Rating Scale than those with CBS-AD (mean [SD], 3.5 [0.5] vs 0.8 [0.8] points/year; *P* = .005).

**Conclusions and Relevance:**

In this cohort study, plasma p-tau217 had excellent diagnostic performance for identifying Aβ or FTP PET positivity within CBS with likely underlying AD pathology. Plasma P-tau217 may be a useful and inexpensive biomarker to select patients for CBS clinical trials.

## Key Points

**Question:**

What is the clinical utility of plasma phosphorylated tau217 (p-tau217) in individuals with corticobasal syndrome (CBS)?

**Findings:**

In this cohort study, plasma p-tau217 was elevated in patients with CBS with positive results on amyloid or tau positron emission tomography (PET), correlated with both biomarkers, and had excellent diagnostic performance in predicting PET positivity. Additionally, p-tau217 identified 2 cohorts within CBS with different clinical characteristics and rates of disease progression, consistent with different underlying etiologies.

**Meaning:**

The findings suggest that plasma p-tau217 accurately differentiated individuals with underlying Alzheimer disease from those with frontotemporal lobar degeneration among patients with CBS, supporting its use as a diagnostic biomarker, including for patient selection in clinical trials for CBS.

## Teaser

This cohort study evaluated the use of phosphorylated tau217 to discern between Alzheimer disease and frontotemporal lobar degeneration among patients with corticobasal syndrome.

## Introduction

Tau protein accumulation is the neuropathological hallmark of several neurodegenerative diseases referred to as tauopathies.^1^ Four-repeat tauopathies (4RT) are primary tauopathies defined by aggregation of tau with 4 repeats of the microtubule binding region,^2,3^ whereas Alzheimer disease (AD) is a secondary tauopathy associated with equal 3R/4R tau accumulation in the presence of amyloid β (Aβ).^4^ To our knowledge, no disease-modifying therapies exist for patients with primary tauopathies (a major unmet medical need),^5^ but lecanemab, an amyloid-clearing antibody, has recently been proposed as a disease-modifying therapy for early amnestic presentations of Alzheimer disease (AD).^6^ Recent clinical trial data^7^ suggest different tauopathies do not respond identically to therapeutics, perhaps due to structural differences in tau aggregates identified by cryoelectron microscopy.^8^ Therefore, for clinical diagnosis, participant selection for clinical trials of tau-directed therapies, and potentially future access to pathology-targeted treatments, identifying the precise underlying neuropathology is important during life.

Several clinical syndromes are classically associated with underlying 4RT, including corticobasal syndrome (CBS), progressive supranuclear palsy Richardson syndrome (PSP-RS), and the nonfluent variant of primary progressive aphasia (nfvPPA).^9,10,11,12,13^ However, the clinical utility of these syndromes to accurately predict an underlying 4R tau proteinopathy varies considerably.^14^ PSP-RS is essentially pathognomonic for 4RT, which has facilitated clinical trials of agents targeting abnormal tau in this population,^5,7,15,16,17^ and apraxia of speech in nfvPPA is strongly associated with 4R-tau pathology.^18,19^ CBS poses a greater diagnostic challenge due to heterogeneous underlying neuropathology. Despite CBS being commonly associated with 4RT and frontotemporal lobar degeneration (CBS-FTLD), up to 40% of clinically diagnosed CBS cases are associated with AD pathology (CBS-AD) without the presence of FTLD.^20,21^ This neuropathological heterogeneity has limited the predictive value of the CBS diagnosis in isolation, which has hindered research efforts, particularly clinical trials of disease-modifying agents targeting neuropathology in CBS.

To improve etiologic prediction in CBS, new biomarkers are needed, preferably ones that are readily available, inexpensive, and noninvasive. Despite technological advances in neuroimaging and positron emission tomography (PET) radiotracers,^22,23^ no 4RT-specific biomarkers, to our knowledge, have yet achieved sufficient diagnostic accuracy to merit clinical use,^24^ although a cerebrospinal fluid biomarker of the microtubule binding region is promising.^25^ For AD, both cerebrospinal fluid and PET biomarkers for Aβ and tau are in use clinically, and these biomarkers have been proposed to refine CBS diagnostic cohorts.^10^ However, despite validation efforts showing excellent differentiation between CBS-AD and CBS-FTLD,^26^ issues with cost, access, and patient acceptance have limited their usefulness in CBS clinical trials. Fortunately, several AD biomarkers have been successfully translated into plasma-based assays, including Aβ peptide ratios and tau phosphorylated at various residues (including threonine181, threonine217, and threonine231).^27,28,29^ Of these, phosphorylated tau217 (p-tau217) may have the best diagnostic accuracy for AD and strongest correlation with Aβ and tau PET^30,31,32^ and therefore may be useful in identifying AD as a primary etiology in CBS.

In this study, plasma p-tau217 was validated against Aβ and tau PET in the 3 most common 4RT syndromes—CBS, PSP-RS, and nfvPPA—in a multicenter longitudinal observational study, the 4R-tauopathy Neuroimaging Initiative (4RTNI). These data were used to determine the optimal diagnostic cutoff for plasma p-tau217 to detect Aβ PET positivity. Then, within CBS, plasma p-tau217 was used to define diagnostic groups to examine differences in clinical and neuroimaging measures of disease progression in patients with likely underlying AD (CBS-AD) vs CBS-FTLD.

## Methods

### Study Design

This retrospective study included research participants presenting between January 2011 and September 2020 at the University of California, San Francisco (UCSF), or at a participating 4RTNI site, including the University of Pennsylvania, Philadelphia; the University of Toronto, Toronto, Ontario, Canada; the University of California, San Diego; Massachusetts General Hospital, Boston; Johns Hopkins University, Baltimore, Maryland; the Mayo Clinic, Rochester, Minnesota; and Columbia University, New York, New York. Follow-up was conducted at 6, 12, and 24 months. All available data for 4RTNI participants were queried (n = 322), and participants with CBS (n = 113), PSP-RS (n = 121), or nfvPPA (n = 39) were selected for inclusion. Participants seen at the UCSF Alzheimer Disease Research Center with typical amnestic AD syndrome and positive amyloid PET results (by expert visual read) were included as positive control individuals (n = 54), and cognitively normal Aβ PET-negative control individuals from 4RTNI (n = 20) were supplemented by inclusion of participants with Aβ PET-negative results in the UCSF Longitudinal Brain Aging Program (n = 39). CBS-AD and CBS-FTLD were defined in later analyses by PET-validated plasma p-tau217 cutoff in patients with CBS with plasma data (n = 83) and a subset with longitudinal magnetic resonance imaging (MRI; n = 51). Participants provided written informed consent at the time of recruitment. The study was approved by the institutional review board of each research site from which participants were recruited. The study followed the Strengthening the Reporting of Observational Studies in Epidemiology (STROBE) reporting guideline.

### Clinical Assessments

All participants underwent a standardized clinical evaluation that included collection of demographic data, structured participant or informant interview, functional assessment, neurological examination, and neuropsychological testing. Blood draw and neuroimaging were typically performed at the same visit as clinical evaluation, and first available data were considered baseline. The primary clinical syndrome was determined based on available data at the time of clinical evaluation by an experienced neurologist or panel of neurologists and neuropsychologists, and followed established diagnostic criteria.^10,11,12^

### Fluid Biomarkers

Nonfasting blood samples were obtained by venipuncture and processed for plasma following Alzheimer Disease Neuroimaging Initiative protocol.^33^ The plasma p-tau217 assay was performed on the Meso Scale Discovery platform, as reported previously.^30^ Operators were blinded to the cohort, and assays were performed in duplicate on the same sample aliquot and processed together in the same batch on a streptavidin small spot plate. All available data were included in the analyses.

### Neuroimaging

MRI data were acquired from multiple centers and scanners; acquisition and processing details are available in the eMethods in Supplement 1. In voxel-based morphometry, a general linear model was fit at each voxel using the Oxford Centre for Functional MRI of the Brain (FMRIB) Software Library version 6.0 (FSL), and all comparisons accounted for age and total intracranial volume.^34^ Familywise error correction was performed using 5000 permutations and threshold-free cluster enhancement.^35^ Voxel-based time trajectories of gray and white matter atrophy were modeled using hierarchical empirical bayesian linear mixed-effects methods.^36^ Annualized atrophy rates were calculated within each group. Atrophy rates were compared between disease group and healthy control individuals after accounting for age and total intracranial volume. In supplemental region of interest analyses, gray matter and white matter volumes were summed in Desikan regions^37^ and grouped by lobe or brainstem. Linear mixed-effect models were used to determine baseline atrophy and annualized atrophy (region of interest × time interaction). The models allowed random intercepts at the individual level and were adjusted for age and total intracranial volume.

For amyloid PET, positivity was defined by expert visual read of PET acquired with carbon 11–labeled Pittsburgh Compound B, [^18^F]florbetapir, or [^18^F]florbetaben tracers, whereas quantitative analyses used Centiloids (CL) calculated using standard methods.^38,39,40^ Tau PET was acquired with [^18^F]flortaucipir (FTP) tracer, and standardized uptake value ratios (SUVR) were calculated from temporal structures using inferior cerebellum as a reference, with SUVR greater than 1.27 defining positivity.^41,42^

### Nonimaging Statistical Analysis

Statistical comparisons were performed with 1-way analysis of variance with pairwise post hoc Bonferroni correction, Pearson χ^2^ test, or Kruskal-Wallis with pairwise post hoc Dunn test and Bonferroni correction when data were not normally distributed. Correlations between biomarkers were calculated using Pearson *R*. Receiver operating characteristic (ROC) analyses determined diagnostic accuracy, and areas under the curve (AUC) and confidence intervals were computed from binary logistic regression. Cutoff values were calculated using Youden indices to maximize sensitivity and specificity.^43^ Linear mixed-effect models were used to evaluate differences in the rate of clinical measure changes over time between clinical syndrome groups. The model allowed random intercepts at the individual level and were adjusted for age, sex, and education. Statistical analyses were done using Stata version 17.0 (StataCorp), SPSS version 28.0.1.0 (IBM), and R version 4.1.1 (R Foundation).

## Results

### Clinical Characteristics

Overall, cohorts were well matched on age, sex, and education (Table). All neurodegenerative cohorts were at a comparable stage of disease severity as measured by duration and Clinical Dementia Rating plus National Alzheimer Coordinating Center FTLD scores.^44^ On the Schwab and England Activities of Daily Living scale, a motor measure of independence for basic function,^45^ individuals with PSP-RS were the most dependent. On the PSP Rating Scale (PSPRS), a severity scale for PSP-related symptomatology, individuals with PSP-RS and CBS had the highest values. A proposed modification of the PSPRS (mPSPRS), focusing on reproducible and clinically relevant PSP-related motor symptomatology,^46^ differentiated the CBS and PSP-RS cohorts. Individuals with AD had the lowest Montreal Cognitive Assessment scores, and all individuals with 4RT were impaired compared with control individuals. Lexical and semantic fluency were lowest in individuals with nfvPPA, and depressive symptomatology on the Geriatric Depression Scale was higher in all neurodegenerative cohorts than in the cognitively normal cohort.

**Table.** Baseline Clinical Characteristics

Characteristic	Mean (SD)						
	CN (n = 59)	AD (n = 54)	CBS (n = 113)	PSP-RS (n = 121)	nfvPPA (n = 39)	CBS-AD (n = 22)	CBS-FTLD (n = 61)
Demographic characteristics							
Age, y	67 (10)	65 (10)^a^	67 (8)	69 (7)	71 (6)^b^	66 (8)	69 (8)
Female, No. (%)	30 (51)	30 (56)	57 (50)	64 (53)	18 (46)	10 (45)	31 (51)
Male, No. (%)	29 (49)	24 (44)	56 (50)	57 (47)	21 (54)	12 (55)	30 (49)
Education, y	18 (2)^a^^,^^b^^,^^c^^,^^d^	16 (4)^e^	16 (4)^e^	15 (4)^e^	15 (4)^e^	17 (3)	16 (4)
Race and ethnicity^f^							
Asian	2 (4)	2 (4)	8 (8)	6 (6)	2 (5)	0 (0)	6 (10)
Black	1 (2)	2 (4)	4 (4)	2 (2)	0 (0)	0 (0)	1 (2)
Hispanic	0 (0)	0 (0)	0 (0)	3 (3)	0 (0)	0 (0)	1 (2)
Native American	0 (0)	0 (0)	1 (1)	0 (0)	0 (0)	0 (0)	0 (0)
White, No. (%)	53 (94)	41 (91)	91 (88)	97 (90)	35 (95)	21 (95)	50 (86)
Clinical measures							
Disease duration, y	NA	5.6 (2.3)	4.8 (3.1)	5.4 (3.2)	4.4 (2.8)	4.7 (2.9)	4.9 (3.3)
CDR + NACC FTLD, global	0.0 (0.1)^a^^,^^b^^,^^c^^,^^d^	1.0 (0.4)^e^	0.9 (0.6)^e^	1.2 (0.7)^e^	1.1 (0.7)^e^	1.0 (0.5)	0.9 (0.5)
CDR + NACC FTLD, box score	0 (0)^a^^,^^b^^,^^c^^,^^d^	6 (3)^e^	4 (4)^d^^,^^e^	6 (3)^c^^,^^e^	5 (5)^e^	5 (3)	4 (3)
SEADL	100 (0)^a^^,^^c^^,^^d^	65 (21)	61 (24)^e^	55 (26)^a^^,^^e^	71 (22)^d^^,^^e^	61 (23)	62 (26)
PSP-RS	1 (2)^c^^,^^d^	NA	25 (12)^a^^,^^e^	33 (15)^a^^,^^e^	11 (9)^c^^,^^d^	19 (10)^g^	28 (12)^g^
mPSPRS	0.2 (0.6)^c^^,^^d^	NA	3.5 (3.2)^a^^,^^d^^,^^e^	7.7 (5.1)^a^^,^^c^^,^^e^	1.0 (1.2)^c^^,^^d^	2.3 (2.4)	3.9 (3.3)
MoCA	28 (2)^a^^,^^b^^,^^c^^,^^d^	17 (6)^a^^,^^c^^,^^d^^,^^e^	22 (6)^b^^,^^e^	22 (4)^b^^,^^e^	22 (6)^b^^,^^e^	21 (5)	22 (6)
D-Words^h^	16 (5)^a^^,^^b^^,^^c^^,^^d^	10 (6)^a^^,^^c^^,^^d^^,^^e^	8 (4)^a^^,^^b^^,^^e^	7 (4)^b^^,^^e^	5 (3)^b^^,^^c^^,^^e^	11 (5)^g^	8 (4)^g^
Animals^i^	23 (6)^a^^,^^b^^,^^c^^,^^d^	12 (6)^e^	13 (6)^d^^,^^e^	11 (5)^c^^,^^e^	10 (6)^e^	14 (7)	13 (6)
GDS-15	1 (1)^a^^,^^b^^,^^c^^,^^d^	3 (2)^c^^,^^d^^,^^e^	4 (3)^b^^,^^e^	6 (4)^b^^,^^e^	4 (4)^e^	5 (3)	4 (4)
AD biomarkers							
P-tau217, pg/mL	0.12 (0.05)^b^^,^^c^	0.72 (0.37)^a^^,^^c^^,^^d^^,^^e^	0.29 (0.33)^b^^,^^e^	0.19 (0.16)^b^	0.18 (0.11)^b^	0.72 (0.38)^g^	0.13 (0.05)^g^
Aβ PET, CL	7 (12)^b^	97 (32)^a^^,^^c^^,^^d^^,^^e^	28 (46)^b^	13 (18)^b^	7 (13)^b^	85 (48)^g^	4 (13)^g^
Tau PET, temporal SUVR	1.13 (0.06)^b^	2.05 (0.44)^a^^,^^c^^,^^d^^,^^e^	1.34 (0.40)^b^^,^^d^	1.12 (0.09)^b^^,^^c^	1.15 (0.08)^b^	1.81 (0.53)^g^	1.15 (0.09)^g^
Aβ+ PET, No./total No. (% positive)	0/59 (0)	54/54 (100)	23/65 (35)	7/41 (17)	6/32 (19)	15/16 (94)	5/40 (13)
Tau+ PET, No./total No. (% positive)	0/18 (0)	50/50 (100)	14/50 (28)	3/41 (7)	4/30 (13)	9/12 (75)	2/26 (7)

Abbreviations: Aβ, amyloid-β; AD, Alzheimer disease; CBS, corticobasal syndrome; CDR, clinical dementia rating scale; CL, Centiloid; CN, cognitively normal; FTLD, frontotemporal lobar degeneration; GDS, Geriatric Dementia Scale; MoCA, Montreal Cognitive Assessment; mPSPRS, modified Progressive Supranuclear Palsy Rating Scale; NA, not applicable; NACC, National Alzheimer Coordinating Center; nfvPPA, nonfluent variant of primary progressive aphasia; p-tau, phosphorylated tau; PET, positron emission tomography; PSP-RS, progressive supranuclear palsy Richardson syndrome; SEADL, Schwab and England Activities of Daily Living; SUVR, standardized uptake value ratios.

^a^ Significant at *P* < .05 vs CN.

^b^ Significant at *P* < .05 vs AD.

^c^ Significant at *P* < .05 vs CBS.

^d^ Significant at *P* < .05 vs PSP-RS.

^e^ Significant at *P* < .05 vs nfvPPA.

^f^ Race and ethnicity data were self-reported by study participants. At the University of California San Francisco Health, race, ethnic group, and ethnicity data are collected from patients using the We Ask Because We Care form. Reporting race and ethnicity in this study was mandated by the National Institutes of Health, consistent with the inclusion of women, minorities, and children policy. Race and ethnicity were collected to assess equity in participation and generalizability of results.

^g^ Significant at *P* < .05 (CBS-AD vs CBS-FTLD).

^h^ D words named in 1 minute as a test of lexical generative fluency.

^i^ Animals named in 1 minute as a test of semantic fluency.

### Quantitative Comparison of AD Biomarkers in 4RT Syndromes

**Figure 1.**
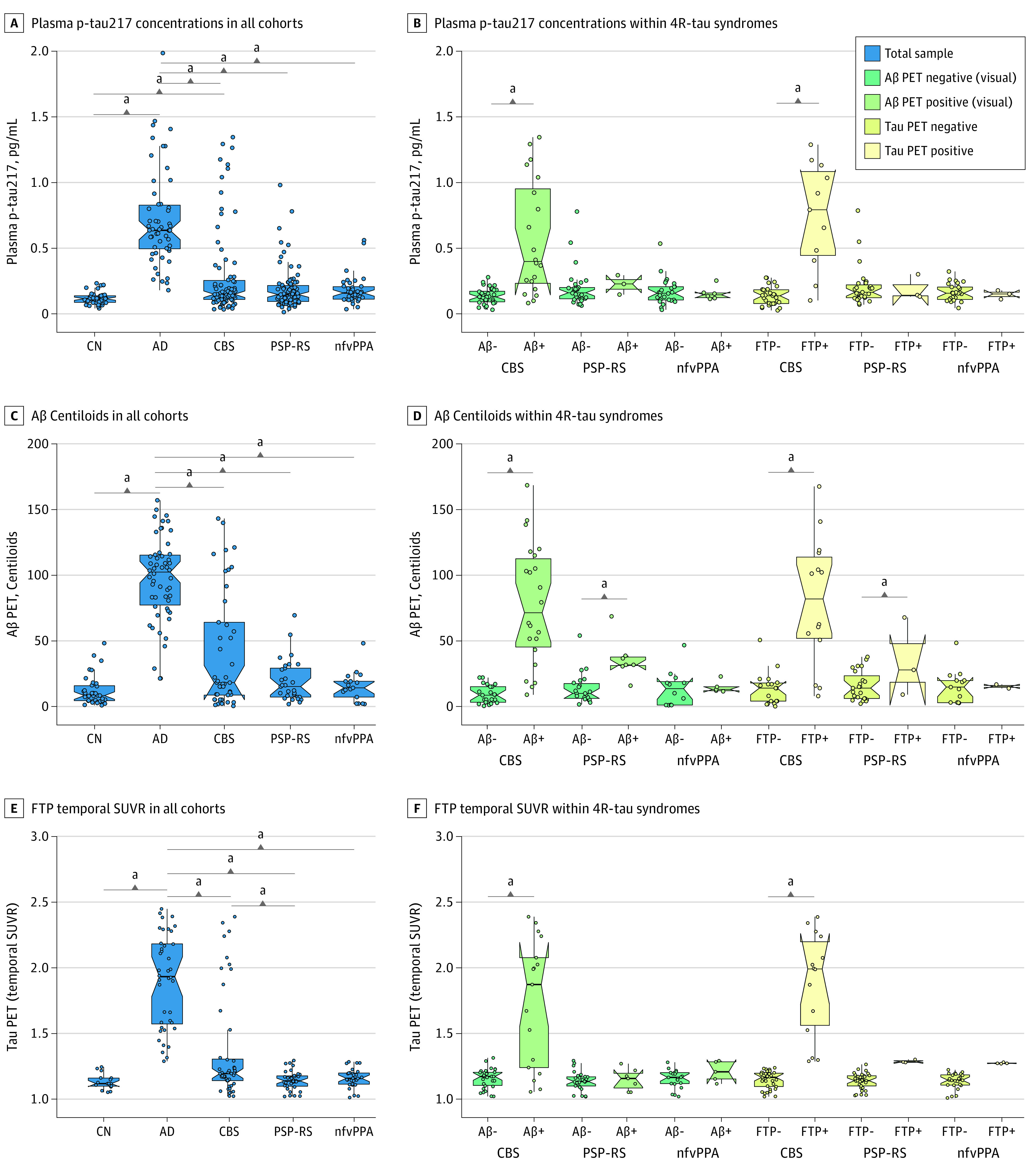
Plasma Phosphorylated Tau217 (P-Tau217) and Positron Emission Tomography (PET) Biomarkers in 4-Repeat (4R)-Tau Syndromes Compared to Alzheimer Disease (AD) and Cognitively Normal (CN) Cohorts Tau PET positivity defined by amyloid-β (Aβ) PET visual read or flortaucipir (FTP) temporal standardized uptake value ratio greater than 1.27. CBS indicates corticobasal syndrome; nfvPPA, nonfluent primary progressive aphasia; PSP-RS, progressive supranuclear palsy Richardson syndrome. ^a^Significant at *P* < .05.

Plasma p-tau217 concentration was highest in individuals with AD compared to those in other cohorts. Those with CBS had more variable p-tau values than those with PSP-RS or nfvPPA and higher p-tau217 than cognitively normal control individuals (Figure 1A; Table). In individuals with CBS, p-tau217 was higher in those with positive Aβ or FTP PET results (mean [SD] CBS-Aβ+, 0.57 [0.43] pg/mL; CBS-Aβ−, 0.14 [0.06] pg/mL; *P* < .001; CBS-FTP+, 0.75 [0.30] pg/mL; CBS-FTP−, 0.14 [0.07] pg/mL; *P* < .001), whereas p-tau217 did not differ by PET status in individuals with PSP-RS (mean [SD] PSP-Aβ+, 0.22 [0.07] pg/mL; PSP-Aβ−, 0.20 [0.15] pg/mL; *P* = .76; PSP-FTP+, 0.19 [0.09] pg/mL; PSP-FTP−, 0.20 [0.15] pg/mL; *P* = .86) or nfvPPA (mean [SD] nfvPPA-Aβ+, 0.16 [0.05] pg/mL; nfvPPA-Aβ−, 0.17 [0.10] pg/mL; *P* = .75; nfvPPA-FTP+, 0.15 [0.03] pg/mL; nfvPPA-FTP−, 0.17 [0.07] pg/mL; *P* = .68) (Figure 1B). Individuals with PSP-RS and nfvPPA had a lower proportion of Aβ and FTP PET positivity compared to those with CBS (Table).

Aβ PET CL values were higher in individuals with AD compared to those in other cohorts (Figure 1C; Table). Individuals with CBS with positive Aβ or FTP PET results had CL values comparable to those with AD (mean [SD] CBS-Aβ+, 75 [49] CL; CBS-Aβ−, 1 [11] CL; *P* < .001; CBS-FTP+, 81 [50] CL; CBS-FTP−, 4 [15] CL; *P* < .001) (Figure 1D), whereas individuals with PSP-RS with visually positive Aβ PET results showed only marginal CL increases relative to individuals with PSP-RS with negative PET results (mean [SD] PSP-Aβ+, 37 [16] CL; PSP-Aβ−, 8 [14] CL; *P* < .001), and no difference was seen for individuals with nfvPPA (mean [SD] nfvPPA-Aβ+, 13 [8] CL; nfvPPA-Aβ−, 6 [14] CL; *P* = .24). FTP temporal SUVR values were elevated in individuals with AD compared to other cohorts (Figure 1E; Table), and in those with CBS-Aβ PET positive results (mean [SD] SUVR, 1.68 [0.51]) compared to those with CBS-Aβ PET negative results (mean [SD] SUVR, 1.15 [0.09]; *P* < .001) (Figure 1F).

### Validation of Plasma P-Tau217 Cutoff for AD PET Biomarkers

**Figure 2.**
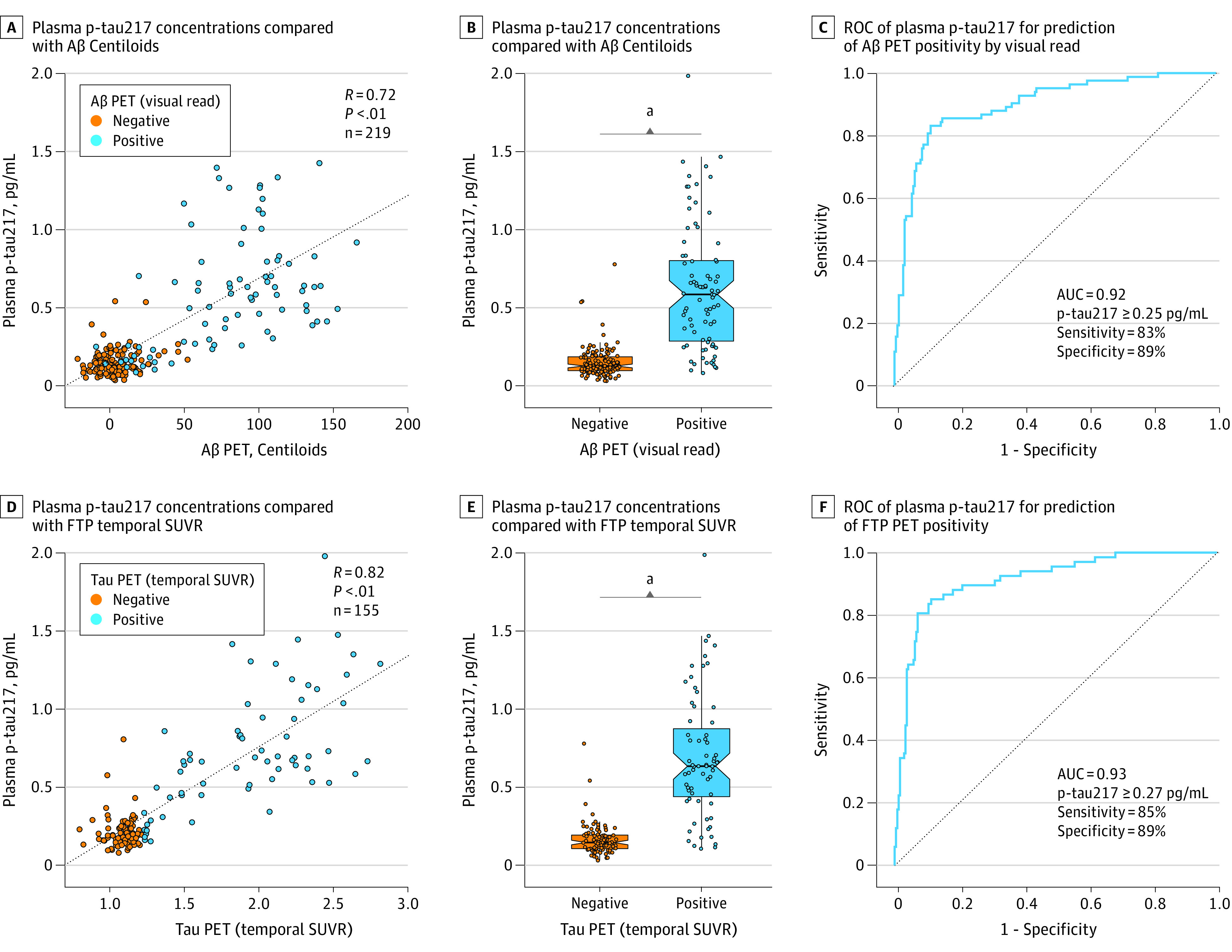
Quantitative Comparison of Plasma Phosphorylated Tau217 (P-Tau217) and Alzheimer Disease (AD) Positron Emission Tomography (PET) Biomarkers and Prediction of PET Positivity Tau PET positivity defined by amyloid-β (Aβ) PET visual read or flortaucipir (FTP) temporal standardized uptake value ratio greater than 1.27. AUC, area under the receiver operating characteristic curve; ROC, receiver operating characteristic curve; SUVR, standardized uptake value ratio. ^a^Significant at *P* < .05.

Plasma p-tau217 was correlated with both Aβ PET CL (*R*, 0.72; *P* < .001) (Figure 2A) and FTP PET temporal SUVR (*R*, 0.82; *P* < .001) (Figure 2D). Among all participants, p-tau217 accurately predicted expert visual read of Aβ PET (AUC, 0.92; 95% CI, 0.88-0.96; *P* < .001) (Figure 2B and C), with an optimal cutoff by Youden Index of 0.25 pg/mL. P-tau217 also accurately predicted temporal SUVR-defined FTP PET positivity (AUC, 0.93; 95% CI, 0.88-0.97; *P* < .001) (Figure 2E and F), with a slightly higher cutoff of 0.27 pg/mL. Within CBS (eFigure 1 in Supplement 1), plasma p-tau217 accurately predicted Aβ and FTP PET positivity (AUC Aβ PET, 0.87; 95% CI, 0.76-0.98; *P* < .001 vs AUC FTP PET, 0.93; 95% CI, 0.83-1.00; *P* < .001), but was less accurate in PSP-RS (AUC Aβ PET, 0.69; 95% CI, 0.41-0.97; *P* = .29 vs AUC FTP PET, 0.63; 95% CI, 0.23-1.00; *P* = .56) and nfvPPA (AUC Aβ PET, 0.50; 95% CI, 0.28-0.72; *P* > .99 vs AUC FTP PET, 0.45; 95% CI, 0.21-0.69; *P* = .78). This difference in predictive ability is consistent with quantitative analyses showing only borderline elevation in PET-positive cases of PSP-RS and nfvPPA (Figure 1D and F).

### Comparison of CBS-AD and CBS-FTLD Defined by Plasma P-Tau217 Concentration

**Figure 3.**
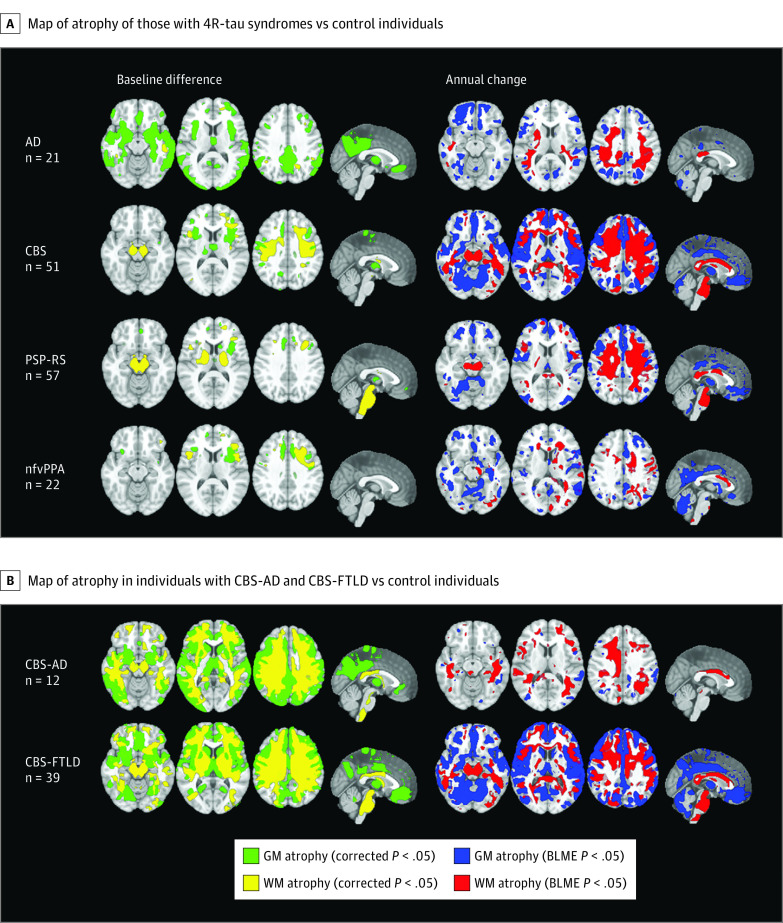
Baseline and Longitudinal Atrophy in 4-Repeat (4R)-Tau Syndromes A, Left panel shows voxel-based morphometry; right panel, bayesian linear mixed effects. B, Left panel shows initial visit; right panel, longitudinal data. All analyses corrected for age and total intracranial volume. AD indicates Alzheimer disease; CBS, corticobasal syndrome; GM, gray matter; FTLD, frontotemporal lobar degeneration; nfvPPA, nonfluent primary progressive aphasia; PSP-RS, progressive supranuclear palsy Richardson syndrome; WM, white matter.

The optimal plasma p-tau217 diagnostic threshold (≥0.25 pg/mL) was used to define CBS-AD and CBS-FTLD in subsequent analyses. At baseline, cross-sectional comparison of these cohorts showed that individuals with CBS-FTLD were more severely impaired on the PSPRS and had lower lexical fluency compared to those with CBS-AD, with no differences on other clinical measures (Table). In the combined CBS cohort, baseline and longitudinal patterns of regional atrophy aligned with prior studies (Figure 3A),^47^ with quantitative region of interest analyses confirming lower baseline volume and increased rates of atrophy in the midbrain and pons in individuals with CBS compared to control individuals and those with AD (eTable 1 in Supplement 1). Baseline voxel-based morphometry analysis comparing individuals with CBS-AD and CBS-FTLD to control individuals showed similar patterns of atrophy overall, with increased posterior cortical atrophy in those with CBS-AD (Figure 3B). Subsequent quantitative region of interest analysis confirmed reduced temporal and parietal lobe volume in individuals with CBS-AD compared to those with CBS-FTLD (eTables 2 and 3 in Supplement 1), especially in the precuneus, consistent with prior work.^47^ Longitudinal bayesian linear mixed-effects analyses within CBS revealed differences in the patterns of volume loss between individuals with CBS-AD and CBS-FTLD (Figure 3B); these were most pronounced in brainstem region of interest analyses, where annual atrophy rates in the midbrain and pons were 1.5 to 2 times higher in individuals with CBS-FTLD than in those with CBS-AD (eTables 2 and 3 in Supplement 1).

**Figure 4.**
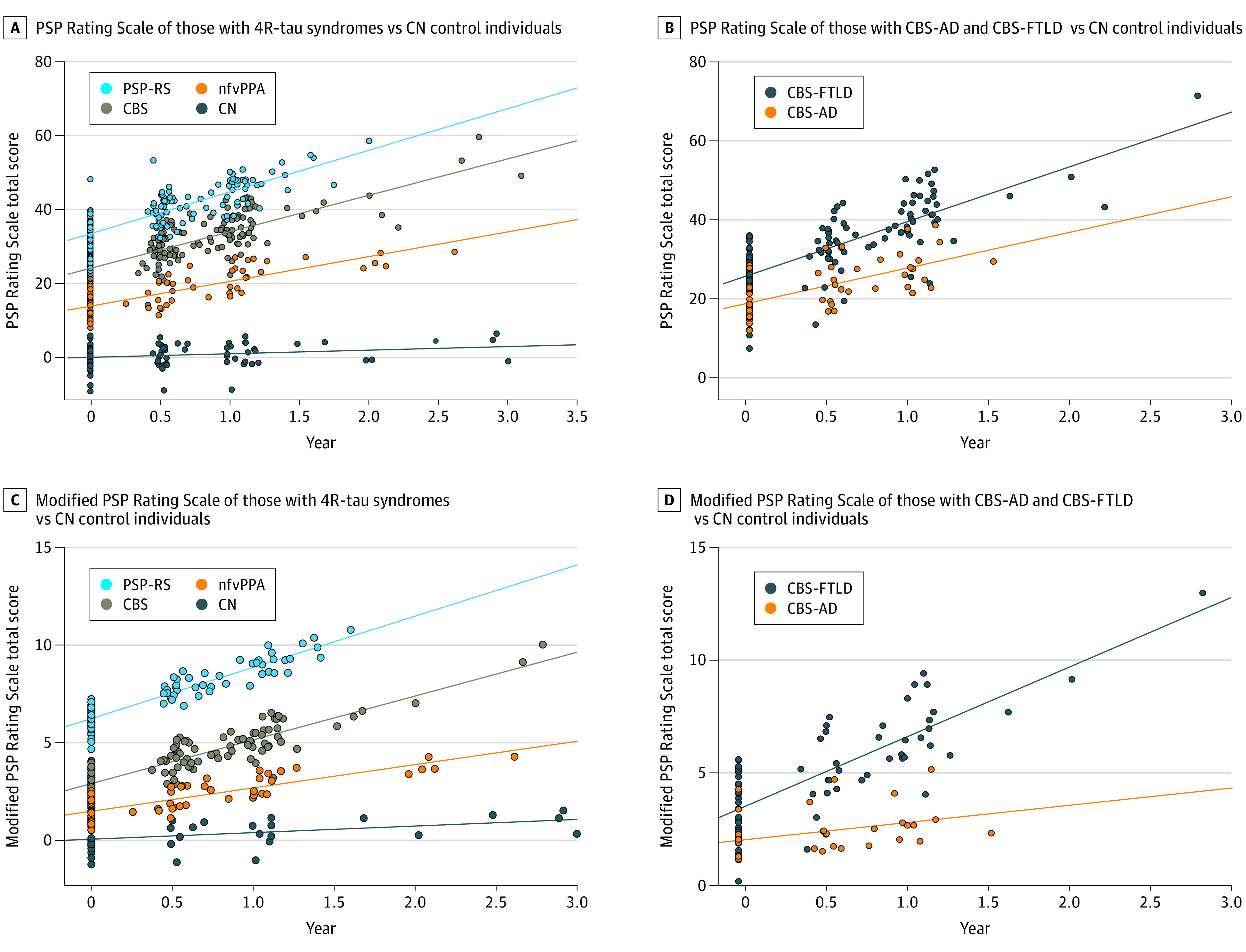
Clinical Trajectories Within 4-Repeat (4R)-Tau Syndromes Predicted values after linear mixed-effect model with syndrome as fixed effect, allowing random slope and intercept, adjusting for age, sex, and education. AD indicates Alzheimer disease; CBS, corticobasal syndrome; CN, cognitively normal; FTLD, frontotemporal lobar degeneration; nfvPPA, nonfluent primary progressive aphasia; PSP-RS, progressive supranuclear palsy Richardson syndrome.

Due to the correlation between disease progression measured on the PSPRS and brainstem atrophy (*R*, −0.53; n = 347; *P* < .001), clinical correlations to the imaging findings within CBS were explored using PSPRS. Baseline PSPRS was higher in individuals with CBS-FTLD than in those with CBS-AD (Table), but no differences were seen in rates of progression longitudinally (Figure 4A and B; eTable 4 in Supplement 1). However, when looking at individual PSPRS domains, which include cognitive and motor subscales, CBS-FTLD progressed faster in the Gait domain (eFigure 2 and eTable 4 in Supplement 1). Interestingly, longitudinal disease progression measured by the mPSPRS, which is focused on PSP-related motor phenomenology, revealed faster progression for individuals with CBS-FTLD compared to those with CBS-AD (Figure 4C and D). No other statistically significant differences were found on other longitudinal clinical measures (eTable 4 in Supplement 1).

## Discussion

In this multicenter longitudinal cohort study, we explored the clinical utility of plasma p-tau217 for detecting AD in individuals with CBS and found plasma p-tau217 was elevated in patients with CBS with positive amyloid or tau PET results and showed excellent diagnostic performance for identifying PET positivity in this cohort. Comparatively, as we have reported previously,^30^ individuals with PSP-RS and nfvPPA showed minimal to no increase in plasma p-tau217, which had less diagnostic utility in these syndromes likely because AD is typically only seen as an incidental copathology, not as primary neuropathology. Using ROC analyses of plasma p-tau217 that showed excellent prediction of Aβ PET positivity within CBS, we derived an optimal cutoff of 0.25 pg/mL to define a diagnostic threshold for CBS-AD vs CBS-FTLD, a cutoff that was reassuringly identical to prior community-based validation studies.^48^ Using this p-tau217 cutoff, we found interrelated differences in clinical and neuroimaging characteristics in CBS-AD and CBS-FTLD, such as faster rates of progression on both the mPSPRS and PSPRS Gait subscale and more prominent longitudinal brainstem atrophy in CBS-FTLD, consistent with our expectations from different protein etiologies.^20,21^

This study was motivated by the need to find new therapies for CBS, as to date only a handful of clinical trials have been conducted in this population. A key challenge for CBS clinical trial development has been the underlying neuropathological heterogeneity, leading to variability in rates of disease progression and uncertainty about which therapies should be selected for investigation. Differentiating CBS-AD from CBS-FTLD would reduce clinical and neuropathological heterogeneity in tau-directed therapeutic trials, as initially proposed in the CBD criteria.^10^ Prior clinical trials enrolling individuals with CBS have used Aβ PET or cerebrospinal fluid for this purpose, but this approach has significant study costs and has prevented participation of centers who lacked access to PET as well as enrollment of participants unwilling to undergo lumbar puncture.^7,49^

The study findings support plasma p-tau217 as a promising alternative to PET and cerebrospinal fluid to detect AD in CBS clinical trials. Specifically, plasma p-tau217 could stratify CBS into CBS-AD and CBS-FTLD to balance treatment arms and evaluate differential treatment response in tau-directed basket trials. Alternatively, one could use plasma p-tau217 to screen for AD as an inclusion criterion to increase diagnostic precision and decrease cost barriers in clinical trials for CBS-AD, perhaps targeting the underlying AD neuropathology. These contexts of use are especially important as we found neuroimaging and clinical differences between individuals with CBS-AD and those with CBS-FTLD both at baseline and longitudinally, with individuals with CBS-AD showing more temporoparietal atrophy at baseline and those with CBS-FTLD having more midbrain and pons atrophy longitudinally. Importantly, these neuroimaging changes correlated with clinical changes measured by the PSPRS, suggesting that the MRI-measured atrophy reflects clinically meaningful end points.^50^ These differing clinical trajectories could potentially be used to increase diagnostic and prognostic confidence in clinical practice as well as to better assess therapeutic efficacy in CBS clinical trials.

In individuals with PSP-RS and nfvPPA, AD is exceedingly rare as the primary neuropathological etiology, but may be present as a copathology in older individuals.^51,52^ Here, we provide quantitative support, as individuals with PSP-RS and nfvPPA had AD biomarker levels similar to age-matched, cognitively-normal, Aβ-negative control individuals. This was true even in cases with a positive Aβ PET results by visual read, where quantitative measures of AD biomarkers were low or borderline compared to Aβ-positive control individuals with AD. These findings suggest that in individuals with PSP-RS and nfvPPA, binary determination of positivity using AD biomarkers may lead to incorrect assumptions about the driving etiology, whereas quantitative approaches may help detect AD copathology and evaluate the contribution of this copathology to the clinical picture. This also highlights the importance of diagnosing the clinical syndrome prior to indiscriminate application of AD biomarkers, as clinical utility differed even between these interrelated neurodegenerative syndromes. Better biomarkers, such as those specific for 4RT, would enable more precise etiological classification in the future.

### Limitations

Our current study has limitations, including unequal diagnostic groups, reliance on PET to define AD, and the lack of a replication cohort. Planned future work will address this gap to validate p-tau217 against autopsy to determine precise contexts of use within 4RT-associated syndromes. Only plasma p-tau217 was included in this study, given the similarity in performance to other phosphorylated isoforms, such as p-tau181,^29^ but isoforms like p-tau231 may have different utility for the milder range of the AD spectrum.^53^ Additionally, inclusion of other plasma biomarkers, such as Aβ ratios,^28^ neurofilament light chain,^32^ and glial fibrillary acidic protein,^54^ may have roles in prognostic stratification or as exploratory outcomes in clinical trials, and future studies should explore the added value of these biomarkers. Additionally, AD as copathology in 4RT is of uncertain clinical significance, so plasma p-tau217 could be used as a research tool to explore the impact of AD copathology in PSP-RS and nfvPPA.

## Conclusions

In this study of individuals with CBS, 1 of the major 4R-tau syndromes, plasma p-tau217 was correlated with both Aβ PET CL and FTP temporal SUVR and accurately predicted both clinically defined Aβ and FTP PET positivity. These findings support the use of plasma p-tau217 as an inexpensive tool in future CBS clinical trials to stratify patients into CBS-AD and CBS-FTLD groups, to improve power to detect treatment effects, or to define populations who may be more responsive to a particular therapeutic modality. Plasma p-tau217 could also be helpful in clinical practice to better estimate CBS rate of disease progression.

## Notes

Supplement 1. eMethods eReferences eFigure 1. Receiver operating characteristics for plasma P-tau217 prediction of PET positivity eTable 1. Baseline Regional Volume and Annualized Atrophy (adjusted for Age and TIV) eTable 2. Baseline Regional Volume and Annualized Atrophy (adjusted for Age and TIV) eTable 3. Baseline Regional Volume and Annualized Atrophy, Compared to Control (%) eTable 4. Adjusted Clinical Characteristics (corrected for Age, Sex, Education) eFigure 2. Clinical trajectories within CBS on PSP Rating Scale subdomains

Supplement 2. Data sharing statement
