# Supplementary material for: Evaluation of Plasma Phosphorylated Tau217 for Differentiation Between Alzheimer Disease and Frontotemporal Lobar Degeneration Subtypes Among Patients With Corticobasal Syndrome
Source: JAMA Neurol. 2023 Apr 3;80(5):495–505. doi: 10.1001/jamaneurol.2023.0488 (PMC10071401; doi:10.1001/jamaneurol.2023.0488)
Supplement: Supplement 1. — eMethods eReferences eFigure 1. Receiver operating characteristics for plasma P-tau217 prediction of PET positivity eTable 1. Baseline Regional Volume and Annualized Atrophy (adjusted for Age and TIV) eTable 2. Baseline Regional Volume and Annualized Atrophy (adjusted for Age and TIV) eTable 3. Baseline Regional Volume and Annualized Atrophy, Compared to Control (%) eTable 4. Adjusted Clinical Characteristics (corrected for Age, Sex, Education) eFigure 2. Clinical trajectories within CBS on PSP Rating Scale subdomains [file jamaneurol-e230488-s001.pdf]

Supplemental Online Content

VandeVrede L, La Joie R, Thijssen EH, et al. Evaluation of plasma phosphorylated tau217 for differentiation between Alzheimer disease and frontotemporal lobar degeneration subtypes among patients with corticobasal syndrome. *JAMA Neurol*. Published online April 3, 2023. doi:10.1001/jamaneurol.2023.0488

eMethods

eReferences

eFigure 1. Receiver operating characteristics for plasma P-tau217 prediction of PET positivity

eTable 1. Baseline Regional Volume and Annualized Atrophy (adjusted for Age and TIV)

eTable 2. Baseline Regional Volume and Annualized Atrophy (adjusted for Age and TIV)

eTable 3. Baseline Regional Volume and Annualized Atrophy, Compared to Control (%)

eTable 4. Adjusted Clinical Characteristics (corrected for Age, Sex, Education)

eFigure 2. Clinical trajectories within CBS on PSP Rating Scale subdomains

This supplemental material has been provided by the authors to give readers additional information about their work.

© 2023 VandeVrede L et al. *JAMA Neurology.*

eMethods

MRI Processing

Before any prepossessing of the images, all T1-weighted images were visually inspected for quality control. Images with excessive motion or image artifact were excluded. T1-weighted images underwent bias field correction using N3 algorithm, and segmentation was performed using SPM12 (Wellcome Trust Center for Neuroimaging, London, UK, http://www.fil.ion.ucl.ac.uk/spm).[1] An intra-subject template was created by non-linear diffeomorphic and rigid-body registration proposed by the symmetric diffeomorphic registration for longitudinal MRI framework.[2] The intra-subject template was segmented also using SPM12's unified segmentation. A within-subject modulation was applied by multiplying the timepoints' jacobian with the intra-subject averaged tissues[3]. A customized group template was generated from the within-subject average gray and white matter tissues and cerebrospinal fluid by non-linear registration template generation using it Large Deformation Diffeomorphic Metric Mapping framework.[4] Modulated intra-subject gray and white matter were geometrically normalized to the group template and then smoothed~(8~mm full width half maximum Gaussian kernel) in the group template. Every step of the transformation was carefully inspected from the native space to the group template. For statistical purposes, linear and non-linear transformations between the group template space and International Consortium of Brain Mapping (ICBM) were applied.

eReferences

1. Ashburner, J. and K.J. Friston, *Unified segmentation.* Neuroimage, 2005. **26**(3): p. 839-51.
2. Ashburner, J. and G.R. Ridgway, *Symmetric diffeomorphic modeling of longitudinal structural MRI.* Front Neurosci, 2012. **6**: p. 197.
3. Ziegler, G., W.D. Penny, G.R. Ridgway, S. Ourselin, K.J. Friston, and I. Alzheimer's Disease Neuroimaging, *Estimating anatomical trajectories with Bayesian mixed-effects modeling.* Neuroimage, 2015. **121**: p. 51-68.
4. Ashburner, J. and K.J. Friston, *Diffeomorphic registration using geodesic shooting and Gauss-Newton optimisation.* Neuroimage, 2011. **55**(3): p. 954-67.

© 2023 VandeVrede L et al. *JAMA Neurology.*

SUPPLEMENTAL FIGURES/TABLES

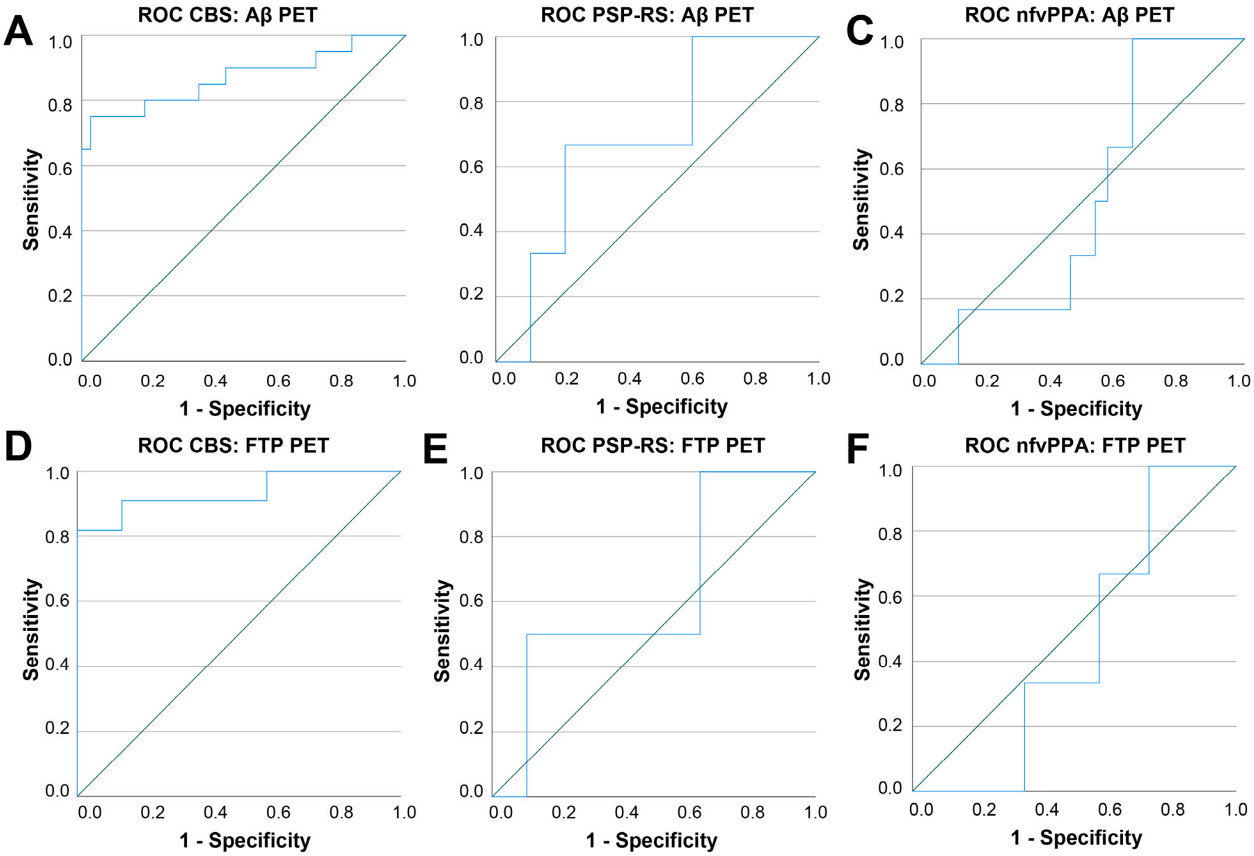

eFigure 1. Receiver operating characteristics for plasma P-tau217 prediction of PET positivity. **(**A,B,C) Prediction of positive Aβ PET by expert visual read for CBS (A), PSP-RS (B), and nfvPPA (C). (D,E,F) Prediction of positive FTP PET, defined as temporal SUVR>1.27, for CBS (D), PSP-RS (E), and nfvPPA (F).

© 2023 VandeVrede L et al. *JAMA Neurology.*

eTable 1. Baseline Regional Volume and Annualized Atrophy (adjusted for Age and TIV)

	CN (n = 38)	AD (n = 21)	CBS (n = 51)	PSP-RS (n = 57)	nfvPPA (n = 22)
Baseline Volume, mean cm^3 (SE)					
Frontal Lobe	272.2 (4.3)[2,3,4,5]	234.2 (5.8)[1]	232.0 (3.7)[1]	237.0 (3.5)[1]	233.2 (5.6)[1]
Cortex	144.0 (2.5))[2,3,4,5]	118.9 (3.4)[1]	124.4 (2.1)[1]	126.0 (2.0)[1]	125.5 (3.3)[1]
White Matter	127.8 (2.1)[2,3,4,5]	115.4 (2.9)[1,3,5]	107.7 (1.8)[1,2]	111.6 (1.7)[1]	106.4 (2.8)[1,2]
Precentral Gyrus	38.7 (0.6)[2,3,4,5]	35.0 (0.9)[1,3]	31.5 (0.5)[1,2,4]	33.7 (0.5)[1,3]	32.9 (0.8)[1]
Temporal Lobe	137.3 (2.1)[2,3,4,5]	109.9 (2.9)[1,3,4,5]	123.5 (1.8)[1,2]	127.5 (1.7)[1,2]	128.5 (2.8)[1,2]
Cortex	88.5 (1.4)[2,3,4,5]	68.4 (1.9)[1,3,4,5]	79.9 (1.2)[1,2]	81.7 (1.1)[1,2]	82.3 (1.8)[1,2]
White Matter	48.6 (0.8)[2,3,4,5]	41.6 (1.1)[1,4,5]	43.7 (0.7)[1,4]	46.1 (0.7)[1,2,3]	45.6 (1.0)[1,2]
Hippocampus	7.67 (0.13)[2,3,4]	6.23 (0.18)[1,3,4,5]	6.99 (0.11)[1,2,5]	7.12 (0.11)[1,2]	7.39 (0.17)[2,3]
Parietal Lobe	172.7 (3.1)[2,3,4,5]	142.6 (4.2)[1,4,5]	146.9 (2.7)[1,4,5]	158.1 (2.5)[1,2,3]	160.2 (4.1)[1,2,3]
Cortex	89.8 (1.6)[2,3,4,5]	69.3 (2.2)[1,3,4,5]	76.7 (1.4)[1,2,3,5]	82.1 (1.3)[1,2,3]	83.4 (2.2)[1,2,3]
White Matter	82.6 (1.5)[2,3,4,5]	73.5 (2.0)[1]	70.3 (1.3)[1,4,5]	76.5 (1.2)[1,3]	75.5 (1.9)[1,3]
Precuneus	30.5 (0.6)[2,3,4,5]	24.5 (0.9)[1,4,5]	26.1 (0.6)[1,4,5]	27.7 (0.5)[1,2,3]	28.3 (0.8)[1,2,3]
Occipital Lobe	73.4 (1.5)[2,3,4]	65.1 (2.0)[1]	67.1 (1.3)[1]	68.0 (1.2)[1]	71.1 (2.0)
Cortex	40.8 (0.8)[2,3,4]	34.2 (1.0)[1,3,4,5]	37.2 (0.7)[1,2]	37.8 (0.6)[1,2]	38.8 (1.0)[2]
White Matter	32.3 (0.6)[3,4]	31.0 (0.8)	29.9 (0.5)[1]	30.7 (0.5)[1]	31.3 (0.8)
Pericalcarine	10.4 (0.3)[3,4]	9.7 (0.4)	9.7 (0.2)[1]	9.7 (0.2)[1]	10.3 (0.3)
Brainstem	19.5 (0.3)[3,4]	19.1 (0.4)[3,4]	17.7 (0.3)[1,2,4,5]	15.9 (0.2)[1,2,3,5]	18.9 (0.4)[3,4]
Midbrain	5.72 (0.09)[3,4,5]	5.45 (0.12)[3,4]	5.08 (0.08)[1,2,4,5]	4.40 (0.07)[1,2,3,5]	5.37 (0.12)[1,3,4]
Pons	12.4 (0.2)[3,4]	12.3 (0.3)[3,4]	11.2 (0.2)[1,2,4,5]	10.1 (0.2)[1,2,3,5]	12.0 (0.3)[3,4]
Atrophy, annual cm^3 change (SE)					
Frontal Lobe	0.95 (0.53)[2,3,4,5]	4.49 (0.84)[1,3,5]	6.96 (0.56)[1,2,4]	4.25 (0.57)[1,3,5]	7.07 (0.82)[1,2,4]
Cortex	0.44 (0.33)[2,3,4,5]	2.35 (0.61)[1]	3.72 (0.40)[1,4]	2.32 (0.41)[1,3]	2.73 (0.55)[1]
White Matter	0.47 (0.33)[2,3,4,5]	2.04 (0.54)[1,5]	3.22 (0.36)[1,4]	1.85 (0.36)[1,3,5]	4.11 (0.52)[1,2,4]
Precentral Gyrus	0.11 (0.09)[2,3,4,5]	0.50 (0.14)[1,3,5]	1.23 (0.09)[1,2,4]	0.78 (0.09)[1,3,5]	1.20 (0.14)[1,2,4]
Temporal Lobe	0.63 (0.25)[2,3,4,5]	3.48 (0.40)[1,4,5]	2.55 (0.27)[1,4]	1.55 (0.27)[1,2,3]	2.21 (0.39)[1,2]
Cortex	0.36 (0.19)[2,3,4]	2.17 (0.35)[1,4,5]	1.82 (0.23)[1]	1.28 (0.23)[1,2]	1.06 (0.32)[2]
White Matter	0.25 (0.10)[2,3,5]	1.26 (0.18)[1,3,4]	0.73 (0.12)[1,2,4]	0.28 (0.12)[1,2,3,5]	1.06 (0.17)[1,4]
Hippocampus	0.04 (0.01)[2,3,4,5]	0.19 (0.02)[1,3,4]	0.16 (0.02)[1,4]	0.11 (0.02)[1,2,3]	0.15 (0.02)[1]
Parietal Lobe	0.70 (0.31)[2,3,4,5]	3.81 (0.52)[1,4]	4.37 (0.34)[1,4]	2.51 (0.35)[1,2,3]	3.33 (0.49)[1]
Cortex	0.30 (0.20)[2,3,4]	2.18 (0.40)[1,4,5]	2.44 (0.26)[1,2,4,5]	1.17 (0.26)[1,2,3]	0.99 (0.35)[2,3]
White Matter	0.32 (0.20)[2,3,4,5]	1.54 (0.35)[1]	1.96 (0.23)[1,4]	1.29 (0.23)[1,3,5]	2.19 (0.32)[1,4]
Precuneus	0.13 (0.05)[2,3,4,5]	0.68 (0.09)[1,4]	0.73 (0.06)[1,4,5]	0.42 (0.06)[1,2,3]	0.51 (0.08)[1,3]
Occipital Lobe	0.32 (0.11)[2,3,4]	1.10 (0.21)[1]	1.22 (0.14)[1,4,5]	0.72 (0.14)[1,3]	0.73 (0.19)[3]
Cortex	0.19 (0.07)[2,3,4]	0.66 (0.20)[1]	0.76 (0.12)[1,5]	0.47 (0.12)[1]	0.27 (0.15)[1,3]
White Matter	0.10 (0.09)[3]	0.36 (0.18)	0.43 (0.12)[1]	0.23 (0.12)	0.44 (0.16)
Pericalcarine	0.05 (0.01)[2,3]	0.12 (0.03)[1]	0.14 (0.02)[1,4]	0.08 (0.02)[3]	0.09 (0.03)
Brainstem	0.05 (0.02)[3,4,5]	0.12 (0.04)[3,4,5]	0.30 (0.03)[1,2,4]	0.37 (0.03)[1,2,3]	0.29 (0.04)[1,2]
Midbrain	0.02 (0.01)[3,4,5]	0.03 (0.01)[3,4,5]	0.10 (0.01)[1,2]	0.12 (0.01)[1,2]	0.11 (0.01)[1,2]
Pons	0.03 (0.02)[3,4,5]	0.07 (0.02)[3,4,5]	0.18 (0.02)[1,2,4]	0.23 (0.02)[1,2,3]	0.19 (0.02)[1,2]

Linear mixed effect model with syndrome as fixed effect, allowing random slope and intercept, adjusted for Age and TIV, with post-hoc pair-wise comparison of estimated marginal mean of baseline ROI volume (syndrome intercept) and longitudinal atrophy (syndrome by time interaction); Superscript denotes significance versus: [1]CN, [2]AD, [3]CBS, [4]PSP-RS, [5]nfvPPA.

© 2023 VandeVrede L et al. *JAMA Neurology.*

eTable 2. Baseline Regional Volume and Annualized Atrophy (adjusted for Age and TIV)

	CBS-AD (n = 12)	CBS-FTLD (n = 39)	*p*
Baseline Volume, mean cm^3 (SE)			
Frontal Lobe	233.9 (8.3)	234.5 (4.5)	0.946
Cortex	125.5 (5.1)	125.9 (2.8)	0.943
White Matter	108.2 (3.8)	108.4 (2.1)	0.975
Precentral	32.3 (1.3)	31.8 (0.7)	0.736
Temporal Lobe	118 (3.8)	126.5 (2.1)	0.05
Cortex	76.8 (2.6)	81.6 (1.4)	0.108
White Matter	41.0 (1.4)	44.8 (0.75)	0.015
Hippocampus	6.75 (0.25)	7.17 (0.14)	0.142
Parietal Lobe	136.1 (5.5)	152.1 (3.0)	0.012
Cortex	69.9 (3.2)	79.8 (1.7)	0.006
White Matter	66.1 (2.9)	72.1 (1.6)	0.069
Precuneus	23.9 (1.1)	27.1 (0.6)	0.012
Occipital Lobe	64.4 (2.9)	68.5 (1.6)	0.208
Cortex	35.3 (1.5)	38.1 (0.8)	0.106
White Matter	28.5 (1.1)	30.3 (0.6)	0.172
Pericalcarine	9.6 (0.5)	9.8 (0.3)	0.651
Brainstem	18.2 (0.6)	17.7 (0.3)	0.521
Cortex	2.76 (0.10)	2.58 (0.05)	0.097
White Matter	15.3 (0.50)	15.1 (0.27)	0.713
Midbrain	5.32 (0.17)	5.07 (0.10)	0.21
Pons	11.6 (0.4)	11.2 (0.2)	0.45
Atrophy, annual mean cm^3 change (SE)			
Frontal Lobe	7.12 (1.28)	6.82 (0.69)	0.838
Cortex	3.55 (1.01)	3.71 (0.54)	0.891
White Matter	3.49 (0.78)	3.17 (0.41)	0.71
Precentral	0.95 (0.22)	1.30 (0.12)	0.156
Temporal Lobe	3.63 (0.70)	2.22 (0.37)	0.075
Cortex	2.51 (0.60)	1.64 (0.31)	0.198
White Matter	1.13 (0.24)	0.62 (0.13)	0.065
Hippocampus	0.20 (0.4)	0.15 (0.02)	0.242
Parietal Lobe	4.99 (0.91)	4.20 (0.48)	0.441
Cortex	2.59 (0.70)	2.41 (0.37)	0.815
White Matter	2.42 (0.54)	1.84 (0.29)	0.349
Precuneus	0.85 (0.16)	0.70 (0.08)	0.386
Occipital Lobe	1.92 (0.34)	1.06 (0.18)	0.025
Cortex	1.09 (0.29)	0.68 (0.14)	0.201
White Matter	0.79 (0.25)	0.34 (0.13)	0.115
Pericalcarine	0.23 (0.05)	0.13 (0.03)	0.084
Brainstem	0.19 (0.06)	0.33 (0.03)	0.045
Cortex	0.06 (0.06)	0.01 (0.03)	0.287
White Matter	0.25 (0.08)	0.32 (0.04)	0.414
Midbrain	0.07 (0.02)	0.11 (0.01)	0.036
Pons	0.10 (0.03)	0.20 (0.02)	0.018

Linear mixed effect model with syndrome as fixed effect, allowing random slope and intercept, adjusted for Age and TIV, with post-hoc pair-wise comparison of estimated marginal mean of baseline ROI volume (syndrome intercept) and longitudinal atrophy (syndrome by time interaction). Highlighted cells denote significance $p<0.05$. TIV, total intracranial volume.

© 2023 VandeVrede L et al. *JAMA Neurology.*

eTable 3. Baseline Regional Volume and Annualized Atrophy, Compared to Control (%)

	AD (n = 21)	CBS (n = 51)	PSP-RS (n = 57)	nfvPPA (n = 22)	CBS-AD (n = 12)	CBS-FTLD (n = 39)
**Baseline Volume, %CN**						
Frontal Lobe	86%	85%	87%	86%	86%	86%
Cortex	83%	86%	88%	87%	87%	87%
White Matter	90%	84%	87%	83%	85%	85%
Precentral Gyrus	90%	81%	87%	85%	83%	82%
Temporal Lobe	80%	90%	93%	94%	86%	92%
Cortex	77%	90%	92%	93%	87%	92%
White Matter	86%	90%	95%	94%	84%	92%
Hippocampus	81%	91%	93%	96%	88%	93%
Parietal Lobe	83%	85%	92%	93%	79%	88%
Cortex	77%	85%	91%	93%	78%	89%
White Matter	89%	85%	93%	91%	80%	87%
Precuneus	80%	86%	91%	93%	78%	89%
Occipital Lobe	89%	91%	93%	97%	88%	93%
Cortex	84%	91%	93%	95%	87%	93%
White Matter	96%	93%	95%	97%	88%	94%
Pericalcarine	93%	93%	93%	99%	92%	94%
Brainstem	98%	91%	82%	97%	93%	91%
Midbrain	95%	89%	77%	94%	93%	89%
Pons	99%	90%	81%	97%	94%	90%
**Annualized Atrophy, %CN**						
Frontal Lobe	473%	733%	447%	744%	749%	718%
Cortex	534%	845%	527%	620%	807%	843%
White Matter	434%	685%	394%	874%	743%	674%
Precentral Gyrus	455%	1118%	709%	1091%	864%	1182%
Temporal Lobe	552%	405%	246%	351%	576%	352%
Cortex	603%	506%	356%	294%	697%	456%
White Matter	504%	292%	112%	424%	452%	248%
Hippocampus	475%	400%	275%	375%	500%	375%
Parietal Lobe	544%	624%	359%	476%	713%	600%
Cortex	727%	813%	390%	330%	863%	803%
White Matter	481%	613%	403%	684%	756%	575%
Precuneus	523%	562%	323%	392%	654%	538%
Occipital Lobe	344%	381%	225%	228%	600%	331%
Cortex	347%	400%	247%	142%	574%	358%
White Matter	360%	430%	230%	440%	790%	340%
Pericalcarine	240%	280%	160%	180%	460%	260%
Brainstem	240%	600%	740%	580%	380%	660%
Midbrain	150%	500%	600%	550%	350%	550%
Pons	233%	600%	767%	633%	333%	667%

Heatmap visualization of results from eTable1, with baseline and annualized atrophy given as a percent comparison to cognitive normal controls for each syndrome. CN, control.

© 2023 VandeVrede L et al. *JAMA Neurology.*

eTable 4. Adjusted Clinical Characteristics (corrected for Age, Sex, Education)

	CBS-AD (n = 18)	CBS-FTLD (n = 58)	*p*
PSPRS			
Baseline	25 (3)	33 (2)	0.02
Annual Progression	9 (3)	15 (2)	0.18
History			
Baseline	5.1 (0.9)	6.2 (0.6)	0.29
Annual Progression	2.0 (0.9)	2.3 (0.6)	0.78
Mentation			
Baseline	2.7 (0.7)	3.1 (0.5)	0.63
Annual Progression	0.9 (0.7)	0.5 (0.5)	0.68
Bulbar			
Baseline	1.2 (0.5)	2.0 (0.3)	0.14
Annual Progression	0.3 (0.7)	0.6 (0.5)	0.69
Ocular Motor			
Baseline	2.1 (1.2)	3.4 (0.9)	0.39
Annual Progression	1.6 (1.8)	4.2 (1.3)	0.25
Limb Motor			
Baseline	7.0 (1.2)	9.1 (0.9)	0.18
Annual Progression	3.4 (1.5)	3.3 (1.1)	0.95
Gait			
Baseline	6.7 (1.9)	9.1 (1.3)	0.33
Annual Progression	3.9 (2.0)	8.8 (1.4)	<0.05
mPSPRS			
Baseline	2.5 (1.0)	4.9 (0.7)	0.06
Annual Progression	0.7 (0.8)	3.4 (0.5)	<0.01
FTLD-CDR			
Baseline	0.9 (0.1)	0.9 (0.1)	0.79
Annual Progression	0.1 (0.2)	0.4 (0.1)	0.17
FTLD-CDR-SB			
Baseline	4.4 (0.8)	3.8 (0.5)	0.51
Annual Progression	0.7 (1.1)	2.3 (0.5)	0.20
SEADL			
Baseline	44 (8)	53 (4)	0.32
Annual Progression	-16 (9)	-17 (4)	0.96
MOCA			
Baseline	17 (2)	20 (1)	0.26
Annual Progression	1 (2)	-2 (1)	0.26
D-Words			
Baseline	8 (1)	8 (1)	0.79
Annual Progression	-3 (1)	-1 (1)	0.24
GDS			
Baseline	4 (1)	5 (1)	0.41
Annual Progression	-1 (2)	0 (1)	0.47

Linear mixed effect model with syndrome as fixed effect, allowing random slope and intercept, adjusted for Age, Sex, and Education, with post-hoc pair-wise comparison of estimated marginal mean of baseline score (syndrome intercept) and longitudinal progression (syndrome by time interaction). Highlighted cells denote significance p<0.05.

© 2023 VandeVrede L et al. *JAMA Neurology.*

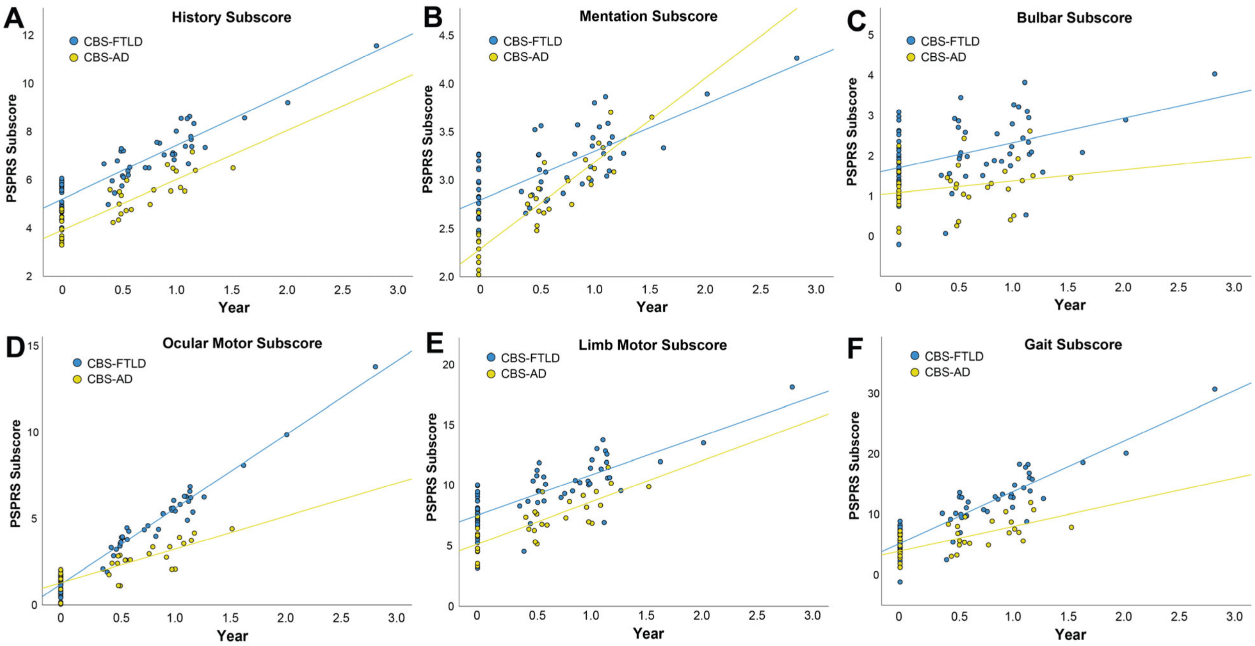

eFigure 2. Clinical trajectories within CBS on PSP Rating Scale subdomains. Predicted values after linear mixed effect model with syndrome as fixed effect, allowing random slope and intercept, adjusting for age, sex, and education.

© 2023 VandeVrede L et al. *JAMA Neurology.*
