# Supplementary material for: Evaluation of Plasma Phosphorylated Tau217 for Differentiation Between Alzheimer Disease and Frontotemporal Lobar Degeneration Subtypes Among Patients With Corticobasal Syndrome
Source: JAMA Neurol. 2023 Apr 3;80(5):495–505. doi: 10.1001/jamaneurol.2023.0488 (PMC10071401; doi:10.1001/jamaneurol.2023.0488)
Supplement: Supplement 2. — Data sharing statement [file jamaneurol-e230488-s002.pdf]

Data Sharing Statement

VandeVrede. Evaluation of Plasma Phosphorylated Tau217 for Differentiation Between Alzheimer Disease and Frontotemporal Lobar Degeneration Subtypes Among Patients With Corticobasal Syndrome. *JAMA Neurol*. Published April 03, 2023.
doi:10.1001/jamaneurol.2023.0488

Data
Data available: Yes
Data types: Deidentified participant data
How to access data: https://ucsf.co1.qualtrics.com/jfe/form/SV_71FzQBhSlHD3ksB
When available: With publication

Supporting Documents
Document types: None

Additional Information
Who can access the data: researchers whose proposed use of the data has been approved
Types of analyses: for a specified purpose
Mechanisms of data availability: after approval of a proposal with a signed data access agreement
